# Isopropyl 4-chloro-3,5-dinitro­benzoate

**DOI:** 10.1107/S160053681004359X

**Published:** 2010-10-30

**Authors:** Xiao-Xi Tai, Jing Sun

**Affiliations:** a Guangdong Food and Drug Vocational College, Guangzhou 510520, People’s Republic of China

## Abstract

In the title compound, C_10_H_9_ClN_2_O_6_, the two nitro groups and the ester group are oriented with respect to the benzene ring at dihedral angles of 49.42 (13)/87.61 (13) and 9.10 (10)°, respectively. In the crystal structure, a weak C—H⋯O inter­action is present. A short Cl⋯O contact of 2.972 (2) Å is also observed in the crystal structure.

## Related literature

For the application of the title compound as a herbicide and fungicide, see: Akira *et al.* (1978 ▶); Ferenc *et al.* (1984 ▶).
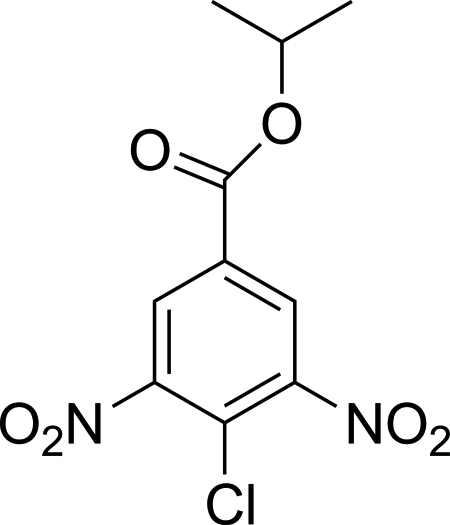


## Experimental

### 

#### Crystal data

C_10_H_9_ClN_2_O_6_
*M* *_r_* = 288.64
Triclinic, 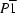

*a* = 4.703 (2) Å
*b* = 10.783 (5) Å
*c* = 12.734 (5) Å
α = 69.483 (12)°
β = 87.75 (2)°
γ = 89.61 (2)°
*V* = 604.3 (5) Å^3^
*Z* = 2
Mo *K*α radiation
μ = 0.34 mm^−1^
*T* = 103 K
0.57 × 0.22 × 0.10 mm

#### Data collection

Rigaku SPIDER diffractometer
Absorption correction: multi-scan (*ABSCOR*; Higashi, 1995 ▶) *T*_min_ = 0.830, *T*_max_ = 0.967
5643 measured reflections
2689 independent reflections
1756 reflections with *I* > 2σ(*I*)
*R*_int_ = 0.030

#### Refinement

*R*[*F* ^2^ > 2σ(*F* ^2^)] = 0.044
*wR*(*F* ^2^) = 0.114
*S* = 1.00
2689 reflections
174 parameters
H-atom parameters constrained
Δρ_max_ = 0.38 e Å^−3^
Δρ_min_ = −0.27 e Å^−3^

### 

Data collection: *RAPID-AUTO* (Rigaku, 2004 ▶); cell refinement: *RAPID-AUTO*; data reduction: *RAPID-AUTO*; program(s) used to solve structure: *SHELXTL* (Sheldrick, 2008 ▶); program(s) used to refine structure: *SHELXTL*; molecular graphics: *SHELXTL*; software used to prepare material for publication: *SHELXTL*.

## Supplementary Material

Crystal structure: contains datablocks I, global. DOI: 10.1107/S160053681004359X/xu5065sup1.cif

Structure factors: contains datablocks I. DOI: 10.1107/S160053681004359X/xu5065Isup2.hkl

Additional supplementary materials:  crystallographic information; 3D view; checkCIF report

## Figures and Tables

**Table 1:** Hydrogen-bond geometry (Å, °)

*D*—H⋯*A*	*D*—H	H⋯*A*	*D*⋯*A*	*D*—H⋯*A*
C5—H5⋯O2^i^	0.95	2.35	3.178 (3)	146

Symmetry code: (i) 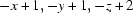.

**Fig. 1.**
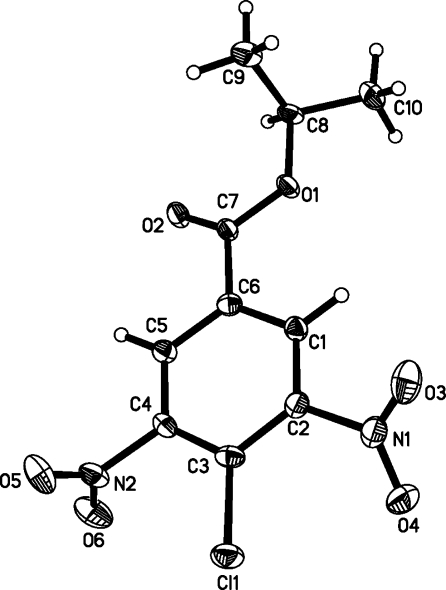
A view of the title compound with the atomic numbering scheme. Displacement ellipsoids were drawn at the 50% probability level.

## supplementary crystallographic information

### Comment

Isopropyl 4-chloro-3,5-dinitrobenzoate (Fig. 1) is a useful herbicide and fungicide (Akira *et al.*, 1978; Ferenc *et al.*, 1984). It was used as the acid compounds to combat fungal diseases and weeds. We report here the crystal structure of the title compound. Two nitro groups (O3/ N1/O4 and O5/N2/O6) attached at C2 and C4, the ester group (O1/C7/O2) attached at C6 form dihedral angles of 49.4 (1)°, 87.6 (1)° and 9.1 (1)° with the mean plane of the C1-benzene ring, respectively. In the crystal structure, adjacent molecules are linked together by the weak C—H···O hydrogen bonds (Table 1).

### Experimental

Commercial isopropyl 4-chloro-3,5-dinitrobenzoate was recrystallized by slow evaporation of methanol solution. Colourless single crystals were formed after several weeks.

### Refinement

H atoms were placed in calculated positions and were allowed to ride on the parent C atoms with C—H distances of 0.95 (aromatic), 0.98 (methyl) and 1.00 Å (methine); *U*_iso_(H) = 1.5U_eq_(C) for methyl H atoms and 1.2U_eq_(C) for the others.

### Crystal data

**Table:**

C_10_H_9_ClN_2_O_6_	*Z* = 2
*M**_r_* = 288.64	*F*(000) = 296
Triclinic, *P*1	*D*_x_ = 1.586 Mg m^−^^3^
Hall symbol: -P 1	Mo *K*α radiation, λ = 0.71073 Å
*a* = 4.703 (2) Å	Cell parameters from 1327 reflections
*b* = 10.783 (5) Å	θ = 3.1–27.5°
*c* = 12.734 (5) Å	µ = 0.34 mm^−^^1^
α = 69.483 (12)°	*T* = 103 K
β = 87.75 (2)°	Prism, colourless
γ = 89.61 (2)°	0.57 × 0.22 × 0.10 mm
*V* = 604.3 (5) Å^3^	

### Data collection

**Table:**

Rigaku SPIDER diffractometer	2689 independent reflections
Radiation source: Rotating Anode	1756 reflections with *I* > 2σ(*I*)
graphite	*R*_int_ = 0.030
ω scans	θ_max_ = 27.5°, θ_min_ = 3.8°
Absorption correction: multi-scan (*ABSCOR*; Higashi, 1995)	*h* = −6→6
*T*_min_ = 0.830, *T*_max_ = 0.967	*k* = −14→13
5643 measured reflections	*l* = −16→15

### Refinement

**Table:**

Refinement on *F*^2^	Primary atom site location: structure-invariant direct methods
Least-squares matrix: full	Secondary atom site location: difference Fourier map
*R*[*F*^2^ > 2σ(*F*^2^)] = 0.044	Hydrogen site location: inferred from neighbouring sites
*wR*(*F*^2^) = 0.114	H-atom parameters constrained
*S* = 1.00	*w* = 1/[σ^2^(*F*_o_^2^) + (0.0496*P*)^2^ + 0.219*P*] where *P* = (*F*_o_^2^ + 2*F*_c_^2^)/3
2689 reflections	(Δ/σ)_max_ < 0.001
174 parameters	Δρ_max_ = 0.38 e Å^−^^3^
0 restraints	Δρ_min_ = −0.27 e Å^−^^3^

### Special details

**Table:**

Geometry. All e.s.d.'s (except the e.s.d. in the dihedral angle between two l.s. planes) are estimated using the full covariance matrix. The cell e.s.d.'s are taken into account individually in the estimation of e.s.d.'s in distances, angles and torsion angles; correlations between e.s.d.'s in cell parameters are only used when they are defined by crystal symmetry. An approximate (isotropic) treatment of cell e.s.d.'s is used for estimating e.s.d.'s involving l.s. planes.
Refinement. Refinement of *F*^2^ against ALL reflections. The weighted *R*-factor *wR* and goodness of fit *S* are based on *F*^2^, conventional *R*-factors *R* are based on *F*, with *F* set to zero for negative *F*^2^. The threshold expression of *F*^2^ > σ(*F*^2^) is used only for calculating *R*-factors(gt) *etc*. and is not relevant to the choice of reflections for refinement. *R*-factors based on *F*^2^ are statistically about twice as large as those based on *F*, and *R*- factors based on ALL data will be even larger.

### Fractional atomic coordinates and isotropic or equivalent isotropic displacement parameters (Å^2^)

**Table:**

	*x*	*y*	*z*	*U*_iso_*/*U*_eq_	
Cl1	1.35262 (13)	0.43193 (6)	0.65836 (5)	0.02487 (18)	
O1	0.4518 (3)	0.85871 (15)	0.76139 (13)	0.0185 (4)	
O2	0.3137 (3)	0.67501 (15)	0.90566 (13)	0.0208 (4)	
O3	1.2776 (4)	0.83028 (19)	0.51714 (14)	0.0338 (5)	
O4	1.1930 (4)	0.66488 (19)	0.46231 (15)	0.0359 (5)	
O5	1.2157 (4)	0.31097 (18)	0.93908 (16)	0.0343 (5)	
O6	0.8756 (4)	0.24121 (18)	0.86381 (17)	0.0364 (5)	
N1	1.1805 (4)	0.7201 (2)	0.53094 (17)	0.0240 (5)	
N2	1.0218 (4)	0.3277 (2)	0.87522 (18)	0.0226 (5)	
C1	0.8350 (5)	0.7223 (2)	0.67783 (18)	0.0170 (5)	
H1	0.7969	0.8119	0.6342	0.020*	
C2	1.0341 (5)	0.6512 (2)	0.64011 (18)	0.0182 (5)	
C3	1.1009 (5)	0.5203 (2)	0.7020 (2)	0.0187 (5)	
C4	0.9569 (5)	0.4649 (2)	0.80468 (19)	0.0170 (5)	
C5	0.7538 (5)	0.5317 (2)	0.8453 (2)	0.0177 (5)	
H5	0.6590	0.4898	0.9161	0.021*	
C6	0.6914 (5)	0.6612 (2)	0.78022 (19)	0.0165 (5)	
C7	0.4638 (5)	0.7312 (2)	0.82410 (19)	0.0160 (5)	
C8	0.2407 (5)	0.9393 (2)	0.7970 (2)	0.0188 (5)	
H8	0.0665	0.8848	0.8302	0.023*	
C9	0.3717 (6)	0.9834 (3)	0.8837 (2)	0.0288 (6)	
H9A	0.4320	0.9057	0.9461	0.043*	
H9B	0.2316	1.0332	0.9117	0.043*	
H9C	0.5372	1.0401	0.8500	0.043*	
C10	0.1679 (6)	1.0513 (2)	0.6922 (2)	0.0290 (6)	
H10A	0.3399	1.1034	0.6591	0.043*	
H10B	0.0261	1.1082	0.7108	0.043*	
H10C	0.0902	1.0155	0.6383	0.043*	

### Atomic displacement parameters (Å^2^)

**Table:**

	*U*^11^	*U*^22^	*U*^33^	*U*^12^	*U*^13^	*U*^23^
Cl1	0.0219 (3)	0.0263 (3)	0.0289 (3)	0.0080 (2)	−0.0003 (2)	−0.0129 (3)
O1	0.0203 (9)	0.0125 (8)	0.0194 (8)	0.0043 (6)	0.0027 (7)	−0.0019 (7)
O2	0.0215 (9)	0.0166 (8)	0.0197 (9)	0.0035 (7)	0.0035 (7)	−0.0011 (7)
O3	0.0334 (11)	0.0378 (12)	0.0238 (10)	−0.0102 (9)	0.0041 (8)	−0.0030 (9)
O4	0.0432 (12)	0.0413 (12)	0.0239 (10)	0.0147 (9)	0.0041 (9)	−0.0130 (9)
O5	0.0284 (11)	0.0266 (10)	0.0409 (11)	0.0075 (8)	−0.0108 (9)	−0.0020 (9)
O6	0.0382 (12)	0.0172 (9)	0.0545 (13)	−0.0016 (8)	−0.0044 (10)	−0.0131 (9)
N1	0.0205 (11)	0.0307 (12)	0.0175 (10)	0.0076 (9)	−0.0001 (8)	−0.0049 (9)
N2	0.0201 (11)	0.0172 (11)	0.0291 (11)	0.0044 (8)	0.0030 (9)	−0.0068 (9)
C1	0.0176 (12)	0.0161 (12)	0.0170 (12)	0.0022 (9)	−0.0039 (9)	−0.0050 (10)
C2	0.0174 (12)	0.0212 (12)	0.0155 (12)	0.0012 (9)	−0.0005 (9)	−0.0057 (10)
C3	0.0149 (11)	0.0200 (12)	0.0248 (13)	0.0037 (9)	−0.0022 (10)	−0.0122 (10)
C4	0.0172 (12)	0.0125 (11)	0.0210 (12)	0.0014 (9)	−0.0049 (9)	−0.0049 (9)
C5	0.0182 (12)	0.0155 (12)	0.0193 (12)	0.0002 (9)	0.0000 (9)	−0.0059 (10)
C6	0.0155 (11)	0.0169 (11)	0.0184 (12)	−0.0007 (9)	−0.0007 (9)	−0.0078 (10)
C7	0.0183 (12)	0.0128 (11)	0.0162 (11)	0.0021 (9)	−0.0039 (9)	−0.0041 (9)
C8	0.0190 (12)	0.0140 (11)	0.0243 (13)	0.0043 (9)	0.0013 (10)	−0.0081 (10)
C9	0.0336 (15)	0.0229 (14)	0.0324 (14)	0.0073 (11)	−0.0029 (12)	−0.0128 (12)
C10	0.0358 (16)	0.0197 (13)	0.0272 (14)	0.0096 (11)	−0.0017 (12)	−0.0029 (11)

### Geometric parameters (Å, °)

**Table:**

Cl1—C3	1.709 (2)	C3—C4	1.384 (3)
O1—C7	1.328 (3)	C4—C5	1.382 (3)
O1—C8	1.475 (3)	C5—C6	1.388 (3)
O2—C7	1.205 (3)	C5—H5	0.9500
O3—N1	1.227 (3)	C6—C7	1.505 (3)
O4—N1	1.217 (3)	C8—C9	1.500 (3)
O5—N2	1.216 (3)	C8—C10	1.503 (3)
O6—N2	1.215 (3)	C8—H8	1.0000
N1—C2	1.472 (3)	C9—H9A	0.9800
N2—C4	1.474 (3)	C9—H9B	0.9800
C1—C2	1.382 (3)	C9—H9C	0.9800
C1—C6	1.388 (3)	C10—H10A	0.9800
C1—H1	0.9500	C10—H10B	0.9800
C2—C3	1.395 (3)	C10—H10C	0.9800
			
C7—O1—C8	116.83 (18)	C1—C6—C7	121.8 (2)
O4—N1—O3	125.7 (2)	C5—C6—C7	117.9 (2)
O4—N1—C2	118.2 (2)	O2—C7—O1	125.9 (2)
O3—N1—C2	116.1 (2)	O2—C7—C6	122.6 (2)
O6—N2—O5	125.8 (2)	O1—C7—C6	111.5 (2)
O6—N2—C4	116.5 (2)	O1—C8—C9	107.98 (19)
O5—N2—C4	117.61 (19)	O1—C8—C10	105.83 (19)
C2—C1—C6	119.1 (2)	C9—C8—C10	113.8 (2)
C2—C1—H1	120.4	O1—C8—H8	109.7
C6—C1—H1	120.4	C9—C8—H8	109.7
C1—C2—C3	122.5 (2)	C10—C8—H8	109.7
C1—C2—N1	117.2 (2)	C8—C9—H9A	109.5
C3—C2—N1	120.3 (2)	C8—C9—H9B	109.5
C4—C3—C2	116.1 (2)	H9A—C9—H9B	109.5
C4—C3—Cl1	120.62 (18)	C8—C9—H9C	109.5
C2—C3—Cl1	123.26 (19)	H9A—C9—H9C	109.5
C5—C4—C3	123.4 (2)	H9B—C9—H9C	109.5
C5—C4—N2	117.8 (2)	C8—C10—H10A	109.5
C3—C4—N2	118.8 (2)	C8—C10—H10B	109.5
C4—C5—C6	118.5 (2)	H10A—C10—H10B	109.5
C4—C5—H5	120.7	C8—C10—H10C	109.5
C6—C5—H5	120.7	H10A—C10—H10C	109.5
C1—C6—C5	120.3 (2)	H10B—C10—H10C	109.5
			
C6—C1—C2—C3	−1.1 (3)	O6—N2—C4—C3	−92.1 (3)
C6—C1—C2—N1	179.8 (2)	O5—N2—C4—C3	87.7 (3)
O4—N1—C2—C1	−131.1 (2)	C3—C4—C5—C6	−0.3 (3)
O3—N1—C2—C1	48.2 (3)	N2—C4—C5—C6	179.6 (2)
O4—N1—C2—C3	49.9 (3)	C2—C1—C6—C5	2.0 (3)
O3—N1—C2—C3	−130.8 (2)	C2—C1—C6—C7	−177.5 (2)
C1—C2—C3—C4	−0.4 (3)	C4—C5—C6—C1	−1.3 (3)
N1—C2—C3—C4	178.6 (2)	C4—C5—C6—C7	178.3 (2)
C1—C2—C3—Cl1	−178.36 (18)	C8—O1—C7—O2	1.9 (3)
N1—C2—C3—Cl1	0.6 (3)	C8—O1—C7—C6	−178.73 (17)
C2—C3—C4—C5	1.2 (3)	C1—C6—C7—O2	170.8 (2)
Cl1—C3—C4—C5	179.18 (18)	C5—C6—C7—O2	−8.8 (3)
C2—C3—C4—N2	−178.78 (19)	C1—C6—C7—O1	−8.7 (3)
Cl1—C3—C4—N2	−0.8 (3)	C5—C6—C7—O1	171.78 (19)
O6—N2—C4—C5	87.9 (3)	C7—O1—C8—C9	84.7 (2)
O5—N2—C4—C5	−92.3 (3)	C7—O1—C8—C10	−153.16 (19)

### Hydrogen-bond geometry (Å, °)

**Table:**

*D*—H···*A*	*D*—H	H···*A*	*D*···*A*	*D*—H···*A*
C5—H5···O2^i^	0.95	2.35	3.178 (3)	146

Symmetry codes: (i) −*x*+1, −*y*+1, −*z*+2.
